# Supplementary material for: Preparation of Chiral Porous Organic Cage Clicked Chiral Stationary Phase for HPLC Enantioseparation
Source: Molecules. 2023 Apr 4;28(7):3235. doi: 10.3390/molecules28073235 (PMC10096354; doi:10.3390/molecules28073235)
Supplement: Supplementary file 1 [file molecules-28-03235-s001.zip › molecules-2296141-supplementary.pdf]

Supplementary Materials

Preparation of Chiral Porous Organic Cage Clicked Chiral Stationary Phase for HPLC Enantioseparation

Ya-Nan Gong, Qi-Yu Ma, Ying Wang, Jun-Hui Zhang *, You-Ping Zhang, Rui-Xue Liang, Bang-Jin Wang *, Sheng-Ming Xie and Li-Ming Yuan

Department of Chemistry, Yunnan Normal University, Kunming 650500, China
* Correspondence: zhangjunhui@ynnu.edu.cn (J.-H.Z.); wangbangjin@ynnu.edu.cn (B.-J.W.)

Experimental Section

1. Synthesis of 2-Hydroxy-1,3,5-benzenetrialdehyde

2-Hydroxy-1,3,5-benzenetrialdehyde was synthesized by *ortho*- and *para*-regioselective formylation of phenol (Figure S1) [S1]. Briefly, phenol (8.3 g, 88 mmol), HMTA (24.1 g, 171.6 mmol), and TFA (75mL) were added into a 250 mL round-bottom flask and heated to reflux at 120 °C under nitrogen atmosphere for 12 h. Afterwards, the reaction temperature was increased to 150 °C and refluxed for another 3 h. The mixture was cooled to 120 °C, hydrochloric acid aqueous solution (100 mL, 3 mol L^{-1}) was added. The mixture was stirred at 100 °C for 30 min and then cooled to room temperature overnight. The resulting yellowish solids were filtered, washed with ethanol and ultrapure water for several times and then dried in a vacuum oven at 70 °C for 10 h. Finally, the crude product was recrystallized with DMSO to obtain pure product (5.0 g). ^{1}H NMR (500 MHz, DMSO-d_6, ppm): δ 10.32 (s, 2H, OH), δ 10.00 (s, 1H, CHO), δ 8.53 (s, 2H, Ar-H); ^{13}C NMR (125 MHz, DMSO-d_6, ppm) δ: 192.14, 191.17, 166.74, 137.77, 128.57, 124.75 (Figure S2).

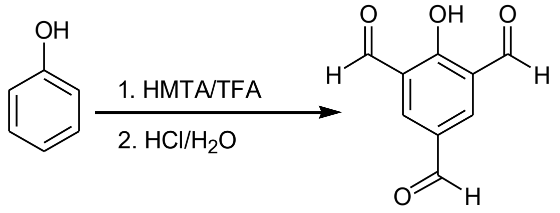

Figure S1. Preparation of 2-hydroxy-1,3,5-benzenetrialdehyde.

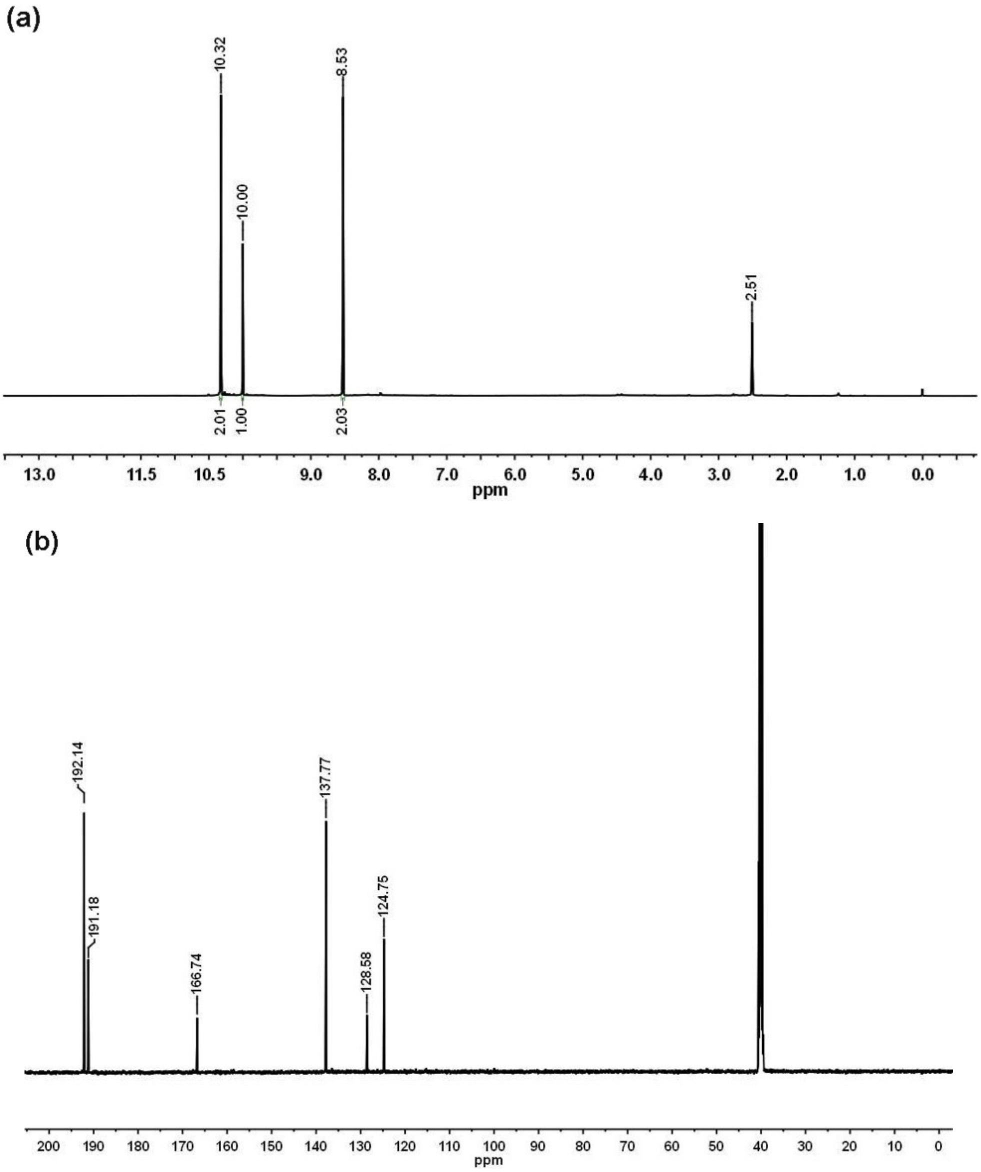

Figure S2. The NMR spectra of 2-hydroxy-1,3,5-benzenetrialdehyde: (a) ^{1}H NMR; (b) ^{13}C NMR.

<table>
<tr><th>Racemates</th><th>This column</th><th>Chiralcel AD-H column</th><th>Chiralpak OD-H column</th></tr>
<tr><td>1-(1-Naphthyl)ethanol</td><td>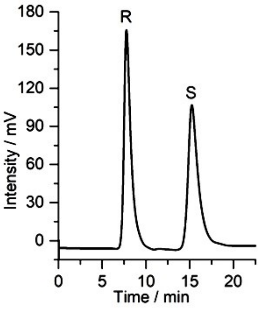
</td><td>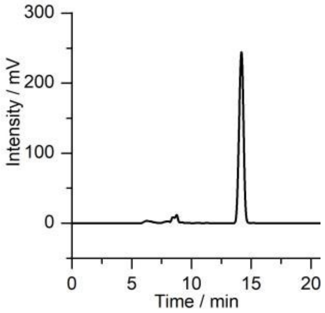
</td><td>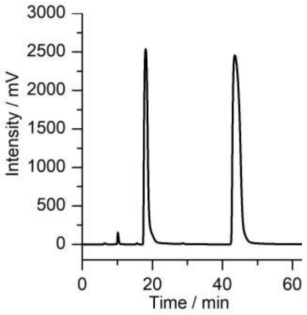
</td></tr>
<tr><td>3-Benzyloxy-1,2-propanediol</td><td>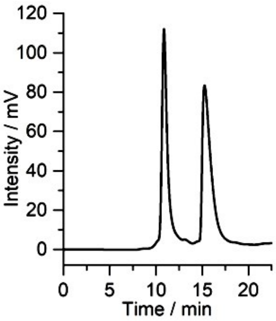
</td><td>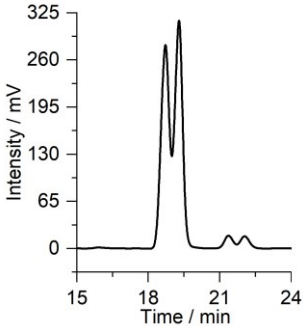
</td><td>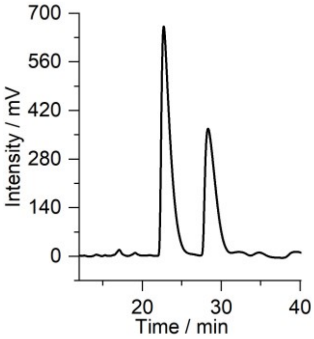
</td></tr>
<tr><td>trans-1,2-Diphenylethylene oxide</td><td>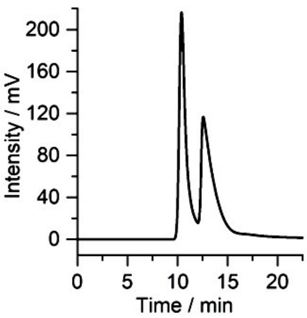
</td><td>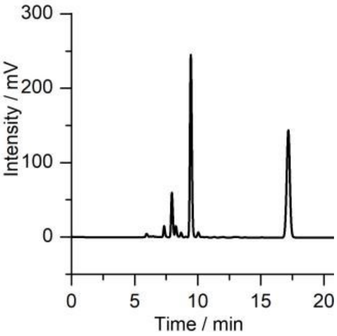
</td><td>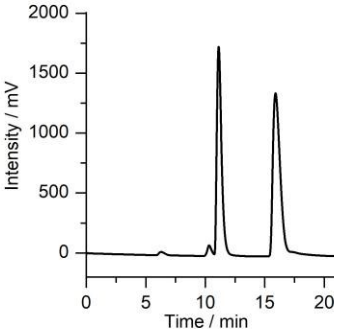
</td></tr>
<tr><td>1-Phenyl-1-propanol</td><td>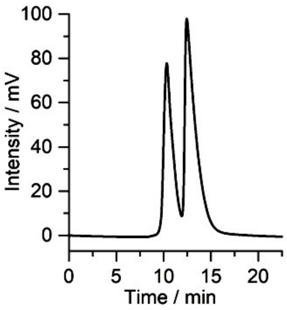
</td><td>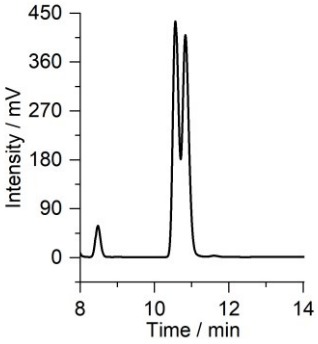
</td><td>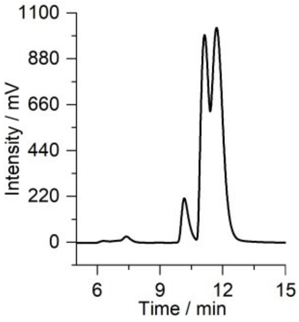
</td></tr>
</table>

Hydrobenzoin	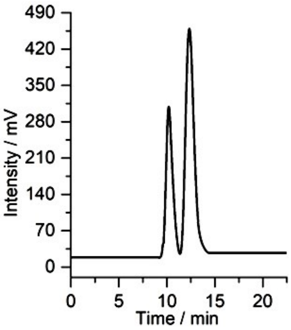	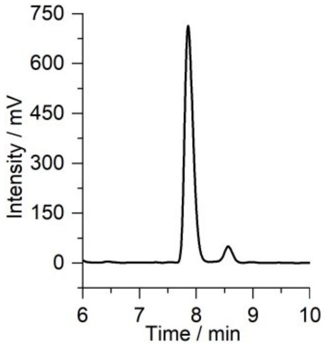	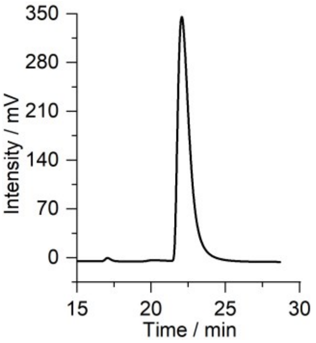
1-Phenylethylamine	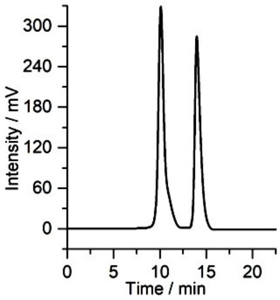	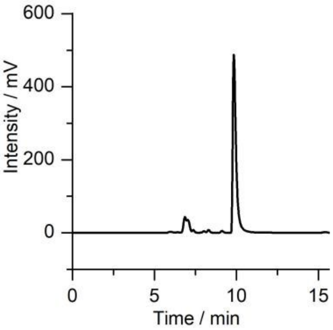	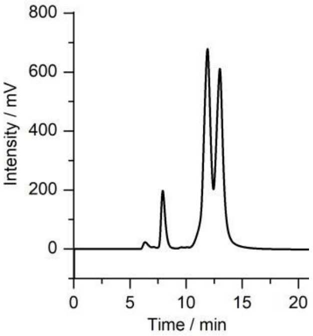
Ofloxacin	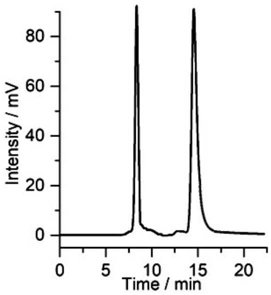	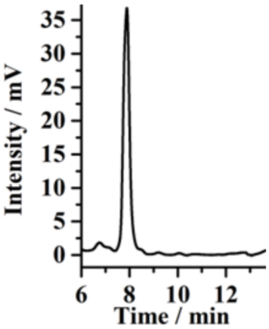	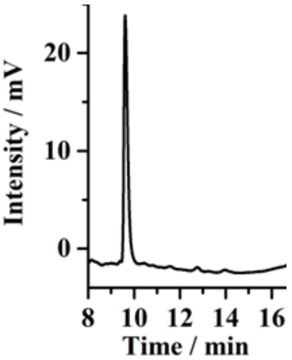

Figure S3. Comparison of the separation of some racemates on this POC-based column, Chiralcel AD-H column and Chiralpak OD-H column.

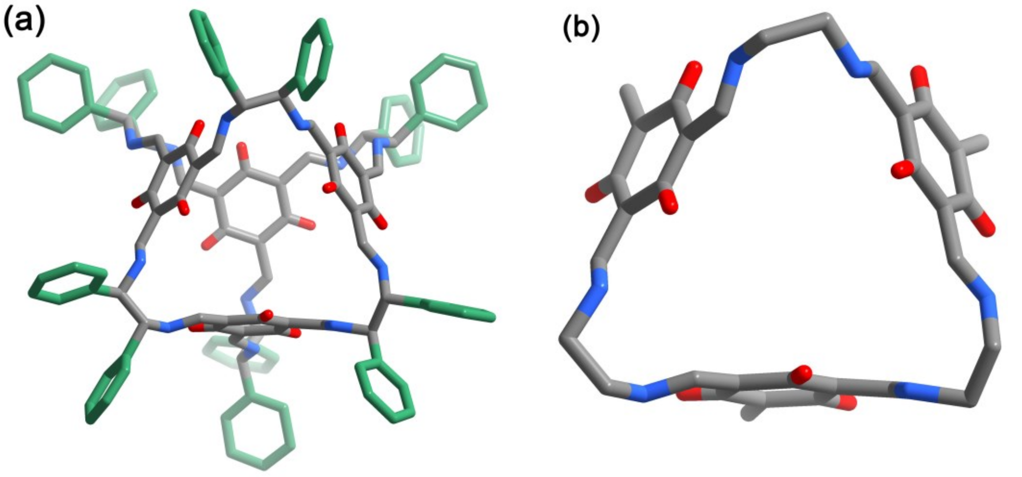

Figure S4. (a) Structure of the chiral POC molecule; (b) shape of the pore window on each face.

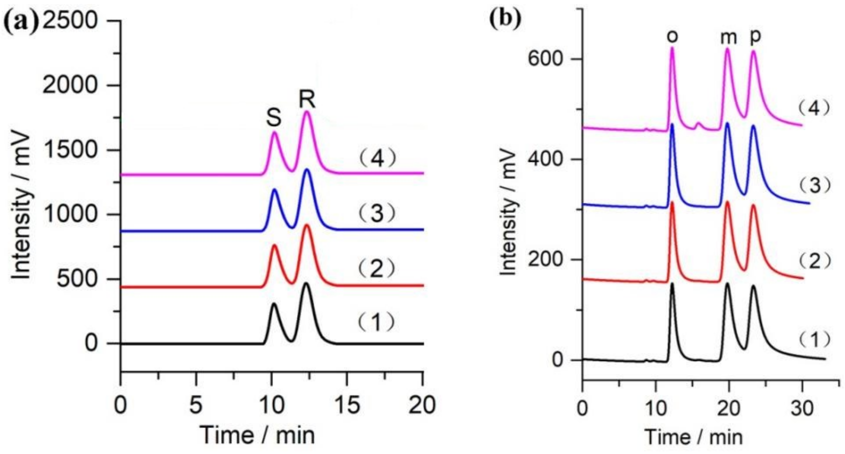

Figure S5. Chromatograms for the separation of (a) hydrobenzoin and (b) iodoaniline after the column were subjected to different injection times. (1)-(4): Chromatograms obtained after the column was undergone 10 injections, 100 injections, 200 injections, and 300 injections, respectively. Other chromatographic conditions are the same as those in Table 1 and Table 2.

Table S1. The results of elemental analysis of SiO_2-SH and CSP.

Analytes	C %	H %	N %
Thiolated silica gel (SiO_2-SH)	3.34	0.87	< 0.05
CSP	13.95	1.67	0.92

References

[S1] Anderson, A.A.; Goetzen, T.; Shackelford, S.A.; Tsank, S.A. Convenient one-step synthesis of 2-hydroxy-1,3,5-benzenetricarbaldehyde. *Synthetic Commun.* **2000**, *30*, 3227-3232.
